# The Influence of Layer Stacking Method on the Mechanical Properties of Honeycomb Skeleton

**DOI:** 10.3390/ma16144933

**Published:** 2023-07-10

**Authors:** Yafei Zhang, Yuqing Zhai, Shiwei Min, Yihua Dou

**Affiliations:** 1 School of Mechanical Engineering, Xi’an Shiyou University, Xi’an 710065, China; zhaiyqxsyu@126.com; 2 Xi’an Key Laboratory of Wellbore Integrity Evaluation, Xi’an Shiyou University, Xi’an 710065, China; 3 Lingyun Technology Group Co., Ltd., Xi’an 710065, China; minswxsyu@126.com

**Keywords:** honeycomb skeleton, tandem structure, pressure-bearing performance, energy absorption

## Abstract

The performance of a multi-layer honeycomb skeleton can be significantly enhanced through tandem connection, while the structure’s properties can be tailored by altering the layer stacking method of the honeycomb skeleton. To investigate the impact of layer stacking methods on the mechanical properties of multilayer honeycomb skeletons, 3D printing technology was used to prepare double-layer honeycomb skeleton tandem structures with different dislocation modes in compression testing. A finite element simulation model was established to conduct quasi-static simulation research. Compared to that of a single-layer honeycomb skeleton, the energy absorption of the honeycomb skeleton tandem structure increased. The optimal bearing capacity of the honeycomb skeleton was achieved when the upper and lower layers were precisely aligned. Once dislocation occurred, both the value of average platform stress and energy absorption decreased. Then, the bearing capacity of the honeycomb skeleton tandem structures increased with an enlargement of the dislocation, reaching its maximum at the half-dislocation period. An increase in the partition thickness and stiffness led to a reduction in the dislocation-induced effects on the mechanical properties. The research results can provide theoretical and data support for the engineering application of honeycomb skeleton tandem structures.

## 1. Introduction

A honeycomb structure is a bionic structure, which is named after its hole shape that is very similar to a bee nest. Because of its high porosity, low mass density, and promising energy absorption characteristics, it has been widely used in aerospace [1], mechanical engineering [2], transportation [3], and other fields. Some examples of this include cushioning and crashworthiness structures in lunar lander systems [4], impact attenuators for cars [5], and packer rubber cylinders in petroleum machinery [6], etc. In the last ten years, scholars at home and abroad have conducted in-depth research on the coplanar and out-of-plane performances of honeycomb compression. According to our previous research, the bearing capacity of a single-layer honeycomb skeleton decreases exponentially with an increase of the height of the cell [7,8], so in practical applications, honeycomb skeleton structures are often used with a tandem of multiple layers. In addition, with an improvement in the compression stroke of its cushioning energy-absorbing structures and the demand for the functional designability of sandwich structures, tandem honeycombs of a multi-layer cellular combination have attracted extensive attention from researchers [9].

The structural parameters of the honeycomb play a decisive role in the mechanical properties of the honeycomb skeleton structure. In recent years, numerous scholars have conducted research on the impact of these structural parameters on the performance of honeycomb skeletons in order to optimize their application characteristics. Zhang et al. [10] carried out a series of plane compression tests on single-layer and double-layer honeycombs, which showed that tandem honeycomb obtains a higher equivalent elastic modulus and collapse stress, and the core assembled with dislocation can achieve almost the same collapse stress with the aligned assembled honeycombs. The length of the dislocation is proportional to the collapse stress. The layer height decides the equivalent modulus and collapse stress of tandem honeycomb, specifically, the dislocation length helps to achieve a higher collapse stress in tandem honeycomb of varying layer heights. Wang et al. [11] conducted a comprehensive axial compression experiment and simulation study on a tandem hexagonal honeycomb structure, discussing the lateral reinforced structure and cellular structure through numerical analyses of the matching effect and cellular effect. The results showed that lateral resistance has significant effect on the deformation mode of the structure, and the mechanical properties of the structure can be obviously improved by filling the tandem honeycomb structure in the porous tube. Based on 3D printing technology, Vijayanand et al. [12] studied the influence of the layer number on the energy absorption of a honeycomb structure, and found that a structure with more honeycomb layers possesses a stronger energy absorption efficiency at the same height. Zhang et al. [13] conducted experimental studies on the out-of-plane properties of honeycomb structures. As a result, buckling, debonding, and fracture have been identified as possible collapse mechanisms. The out-of-plane strength of honeycomb is less related to the cell geometry and highly sensitive to the density of the honeycomb. Liu et al. [14], inspired by the way in which bamboo nodes and nodal diaphragms enhance the transverse strength of bamboo, modified a non-convex multi-corner thin-walled column by adding bulkheads in the column. The results indicated that the strength improvement in the hollow-core structures was significant. The impact resistance of honeycomb sandwich structures has also been a focus of research, in view of the wide range of application scenarios for honeycomb skeletons. Research on the impact resistance of multilayer honeycomb structures has been helpful for mastering the failure behavior and damage mode of these structures [15]. Chen et al. [16] studied the influence of the number of layers of a honeycomb sandwich panel on the impact resistance of a projectile, and found that increasing the number of layers can effectively reduce the contact stress between a projectile and honeycomb sandwich panel, prolonging the interaction time between them. According to Yoshiaki’s [17] research, the energy absorption efficiency and energy absorption capacity of pyramid multilayer honeycomb are better than those of uniform multilayer honeycomb under an impact load. Sun et al. [18] conducted a numerical study on the dynamic deformation pattern, platform stress, and energy absorption performance of a multi-layered regular arrangement with circular honeycombs, and revealed the effects of the configuration parameters and impact velocity on the deformation results. Based on the concept of gradient material, Yao et al. [19] changed the strength of the tandem honeycomb in a structure into a gradient distribution. The results showed that the improved gradient structure had a better eccentric stability and energy absorption effect.

Honeycomb skeleton construction requires the utmost consideration of its material selection to achieve an optimal performance [20]. Paper and aluminum honeycomb are the most commonly used materials. Zhou et al. [21] studied the mechanical properties and energy absorption properties of different types of double-layer Nomex honeycombs through experiments. The experimental research pointed out that the double-layer Nomex honeycomb structure with an interlayer could effectively bear a large number of collapse forces and had a wide range of applications in the field of anti-collision. On the contrary, the double-layer Nomex honeycomb structure without an interlayer was more suitable for reducing the initial failure stress, which made it an excellent damping and energy absorption structure. Weng et al. [9] used a Nomex honeycomb horizontal pressure experiment to find that honeycomb skeleton tandem structures have better pressure-bearing and energy absorption characteristics than single-layer honeycomb structures. Fazilati et al. [22] used a genetic algorithm to design and optimize the multi-layer structure of a hexagonal metal honeycomb energy absorber. The results indicated that the energy absorption performance could be improved by adopting a multi-layer structure and increasing the number of layers. Fan et al. [23] carried out a compressive experiment on single-layer, double-layer (without an aluminum interlayer), and double-layer (with an aluminum interlayer) aramid paper honeycombs. It was found that the energy absorption characteristics of the double-layer paper honeycomb were better, and adding an aluminum interlayer could improve the energy absorption characteristics of this double-layer paper honeycomb. Li et al. [24] carried out simulation and experimental studies on the static compression in tandem aluminum honeycomb, with the results showing that tandem honeycomb structures can absorb more energy than single honeycomb structures. In order to improve the buffer performance of a walkable lunar lander, Zhou et al. [25] developed an optimization method for the honeycomb structure of tandem aluminum, assessing the buffer energy absorption properties through numerical simulations and experiments. They mutually confirmed that the honeycomb skeleton of the tandem had excellent mechanical and buffer energy absorption properties. Lin et al. [26] studied the mechanical behavior of multi-layer aluminum honeycomb without a separator under an out-of-plane compression load, and found that the energy absorbed by the multi-layer aluminum honeycomb was higher than that of single-layer aluminum honeycomb under the same compression displacement. Giulia et al. [27] analyzed the energy absorption capacity of single-layer and double-layer aluminum honeycomb sandwich structures through low-speed impact experiments, evaluated the impact absorption mechanism through computed tomography images and visual inspection, and preliminarily confirmed the existence of a size effect. Zhao et al. [28] simulated the dynamic response of double-layer aluminum honeycomb under an explosion load through experiments and numerically simulates. The research showed that, when the side lengths of upper and lower honeycomb cells are the same, the anti-explosion performance is the best when the relative density of the upper and lower honeycomb structures is 3:1.

The aforementioned studies primarily focused on aluminum and paper honeycomb structures, with only a limited number of investigations being conducted on rubber honeycomb structures. Rubber materials are extensively utilized in mechanical devices for cushioning and damping purposes, such as automobiles, due to their exceptional elasticity that enables them to regain their original shape after undergoing elastic deformation. With the advancement of modern manufacturing technology, the range of rubber products is becoming increasingly diverse. If rubber is utilized in the preparation of honeycomb skeleton structures, the resulting material will exhibit viscoelastic properties while retaining the advantageous high specific strength of the honeycomb structure. This structural material can rebound after deformation and overcome the limitation of single use associated with traditional honeycomb structures when they are used as energy-absorbing materials. Due to the low rigidity of rubber material, it is prone to deformation. Therefore, if a single-layer honeycomb structure is used, its pressure-bearing performance will be greatly reduced. To guarantee its capacity to withstand pressure, the rubber honeycomb must be applied in a multi-layer combination series. However, there have been few studies on the multi-layer combination series of rubber honeycomb skeleton structures, and theory is urgently needed to guide the application of these rubber honeycomb skeleton series structures.

In this paper, a thermoplastic polyurethanes (TPU) rubber honeycomb skeleton tandem structure was prepared using 3D printing technology. Through experiments and finite element simulations, the effects of the dislocation distance, dislocation angle of the upper and lower honeycombs, and interlayer thickness and material on the mechanical properties of the honeycomb skeleton tandem structure under different dislocation modes (translation and rotation) were investigated.

## 2. Materials and Methods

### 2.1. Modeling

**Figure 1:**
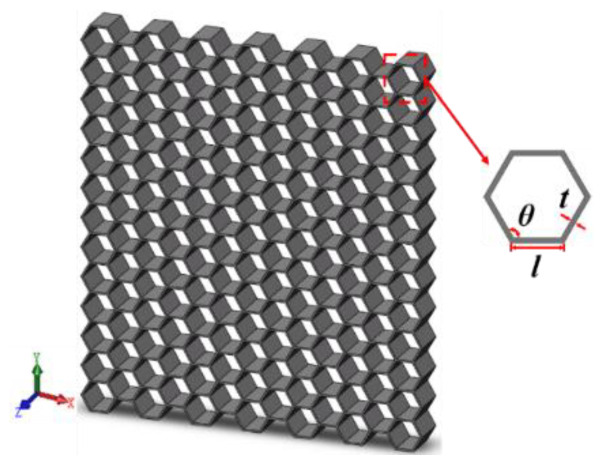
Schematic diagram of geometric structure of honeycomb.

In this paper, the most widely used regular hexagon was selected as the honeycomb cell structure and the influence of the tandem structure of a honeycomb skeleton was studied. A hexagonal honeycomb geometric structure has three dimensions: length (*X*), width (*Y*), and thickness (*Z*) [29]. For a single honeycomb cell, it can be characterized by three main geometric parameters: wall thickness (*t*), side length (*l*), and inner flat angle (*θ*), as illustrated in Figure 1.

**Figure 2:**
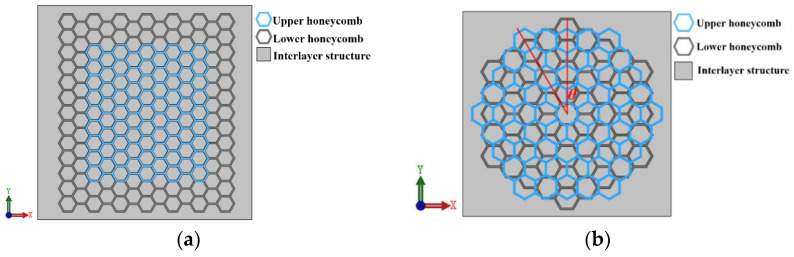
Schematic diagram of honeycomb skeleton tandem structure model: (**a**) translation dislocation; and (**b**) rotational dislocation.

This article examines the impact of translation and rotation dislocations on the mechanical properties of honeycomb structures. The tandem structure of a honeycomb skeleton consists of upper honeycomb and lower honeycomb. When studying translation dislocation, the tandem structure of a honeycomb skeleton is shown in Figure 2a.

The translation dislocation of a honeycomb skeleton tandem structure can be divided into two cases: *X*-axis dislocation and *Y*-axis dislocation. When rotation dislocation is studied, the tandem structure of a honeycomb skeleton is shown in Figure 2b. Due to the time and labor required to print test models, two research methods—experimentation and numerical simulation—were employed to determine the influence of these dislocation modes on the mechanical properties of honeycomb skeletons. In the test condition, when there was *X*-axis dislocation, the dislocation distances were 0,  3l/4, and 3l/2, respectively. In the numerical simulation condition, the dislocation distances were 0, 3l/8, 3l/4, 9l/8, and 3l/2, respectively. When there was *Y*-axis dislocation, the dislocation distances were 0, $\sqrt{3}l/4$, and $\sqrt{3}l/2$, respectively. In the numerical simulation condition, the dislocation distances were 0, $\sqrt{3}l/8$, $\sqrt{3}l/4$, $3\sqrt{3}l/8$, and $\sqrt{3}l/2$, respectively. When there was a rotating misalignment, the misalignment angles were 0°, 10°, 20°, and 30°, respectively. In the numerical simulation condition, the misalignment angles were set as 0°, 10°, 20°, and 30°, respectively.

### 2.2. Experimental Method

Using a JGAURORA A6 3D printer, TPU rubber was used as a consumable to prepare experimental compression samples of a honeycomb skeleton. The printing accuracy could reach the residual of 0.05 mm, and the TPU rubber material parameters are shown in Table 1.

**Figure 3:**
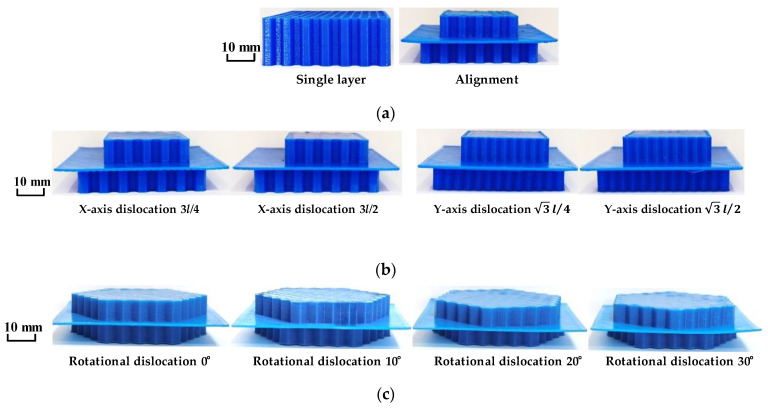
Compression test specimen: (**a**) single-layer and alignment honeycomb model; (**b**) translational dislocation honeycomb model; and (**c**) rotating misaligned honeycomb model.

The cell parameters of the compressed specimen honeycomb skeleton were set as follows: wall thickness *t* = 0.3 mm, side length *l* = 3 mm, and internal flat angle *θ* = 120°. The total heights of the samples were consistent, all of which were 12 mm. The experimental compression samples were prepared as shown in Figure 3.

**Figure 4:**
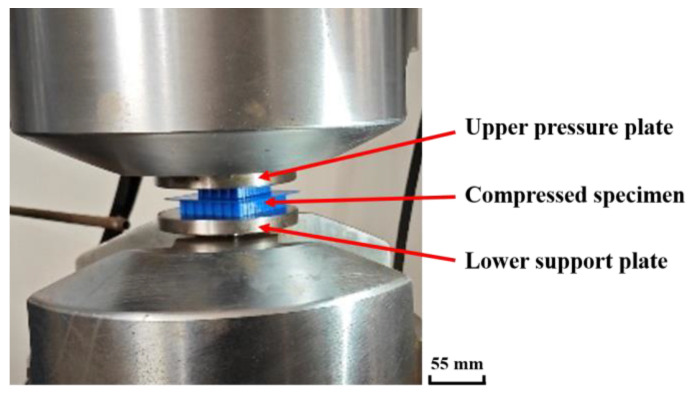
Clamping mode of compression test sample of honeycomb skeleton tandem structure.

The compression test of the honeycomb skeleton tandem structure was carried out on a PLD-300 fatigue test bench and the clamping mode of the samples is shown in Figure 4.

The compressed specimen was placed on the lower support plate of the fatigue testing machine and the pressure plate of the fatigue testing machine was controlled by LETRY code to maintain a constant compression speed of 1 mm/min downward. Under this condition, the strain rate of the tandem structure of the honeycomb skeleton was 0.00167 $s^{-1}$, and it was considered to be a quasi-static compression process of the tandem structure of the honeycomb skeleton.

In the experiment, the computer-controlling terminal of the fatigue testing machine automatically monitored the displacement value H and contact pressure F of the pressure plate in the fatigue testing machine synchronously. The definition of the average contact stress on the upper surface of the sample is:

(1) $$\bar{\sigma }=\frac{F}{S},$$

where F is the contact pressure of the pressure plate on the fatigue testing machine, N. S is the contact area between the honeycomb compression specimen and the pressure plate of the fatigue testing machine, mm^2^.

The definition of the compression ratio of the sample is:

(2) $$\varepsilon =\frac{H}{h},$$

where H is the displacement value of the pressure plate in the fatigue testing machine, mm. h is the height of the sample, mm.

### 2.3. Numerical Simulation Method

According to the actual size of the honeycomb structure, the finite element model was established. The material of the honeycomb skeleton basal body was TPU rubber. The material density was 1160 kg/m^3^, the elastic modulus was 17.3 MPa, and the Poisson’s ratio was 0.47 [31]. The upper and lower rigid plates were set as structural steel, of which the density was 2700 kg/m^3^, the elastic modulus was 70 GPa, and the Poisson’s ratio was 0.33 [32].

Honeycomb compression is a very complex process, which involves multiple nonlinear problems of structure and material. In order to avoid the error of the non-convergence of the model and improve its calculation speed, ABAQUS/Explicit solver was used for the calculation in the present work. The honeycomb skeleton tandem structure was located between two plates, in which the lower support plate was set as a fixed constraint and the upper pressure plate was compressed downward along the axial direction with a compression speed of 1 mm/min. In order to avoid model penetration, penalty function and hard contact were used to define tangential and normal interaction, and the friction coefficient of self-contact was set to 0.3. Mesh was generated with hexahedral elements.

### 2.4. Model Verification

**Figure 5:**
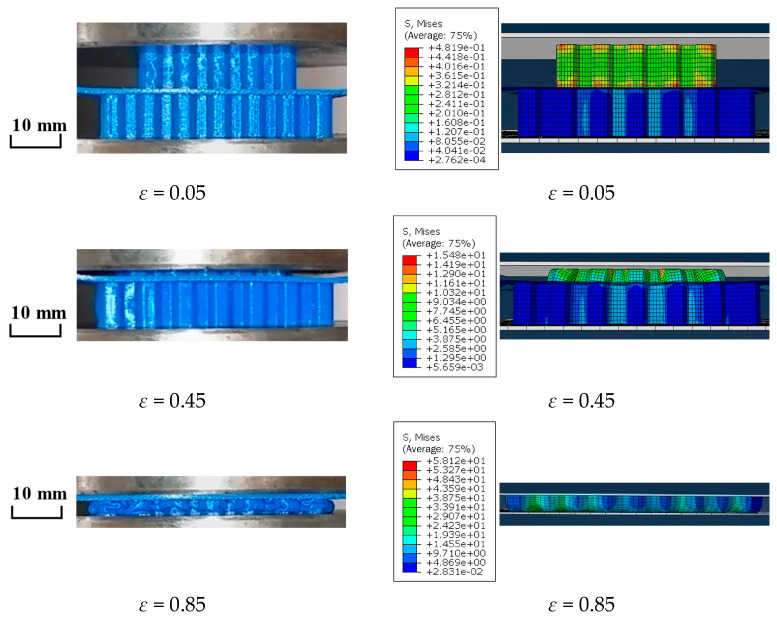
Comparison between compression experiment and finite element deformation of honeycomb skeleton.

Figure 5 shows a deformation comparison diagram of honeycomb skeleton tandem structures under the same conditions between the experiment and numerical simulation with the same compression distance.

It can be seen from the figures that the deformation modes of the two were consistent.

**Figure 6:**
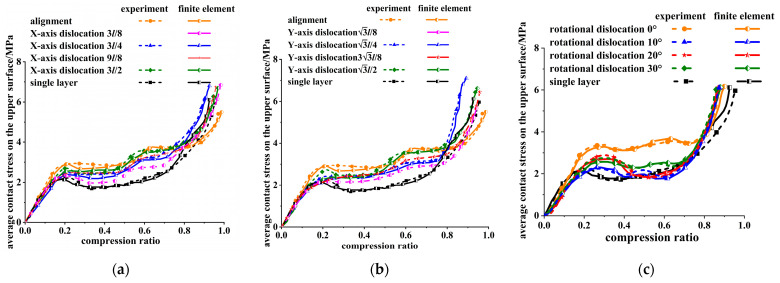
Relationship between average contact stress and compression ratio of upper surface of honeycomb skeleton under different dislocation modes: (**a**) dislocation along the *X*-axis; (**b**) dislocation along the *Y*-axis; and (**c**) rotation dislocation.

The average contact stress and compression ratio of the upper surface were extracted during the compression process of each honeycomb skeleton from Figure 5, and plotted in Figure 6.

The relative error $E_{\bar{P}}$ is defined as the average error of each point between the experimental and finite element curves.

(3) $$E_{\bar{p}}=\frac{\frac{P_{E1}-P_{F1}}{P_{E1}}+\frac{P_{E2}-P_{F2}}{P_{E2}}+\cdots +\frac{P_{En}-{P_{F}}_{n}}{P_{En}}}{n},$$

where: $E_{\bar{P}}$ is the relative error, dimensionless; $P_{E}$ is the experimental value of the average contact pressure on the upper surface of the honeycomb skeleton, MPa; $P_{F}$ is the simulation value of the average contact pressure on the upper surface of the honeycomb skeleton, MPa; and *n* is the sample amount, dimensionless.

Formula (3) is used to calculate the relative error between the experiment and the finite element curve, and the results are summarized in Table 2.

It can be seen from Table 2 that the maximum error of the translation misalignment was 5.63%, the maximum error of the rotational dislocation was 4.61%, and the error of single-layer honeycomb was 6.21%. This shows that the finite element simulation accurately simulated the experimental compression process, and confirms the effectiveness of the established finite element model.

## 3. Deformation Mode of Honeycomb Skeleton

**Figure 7:**
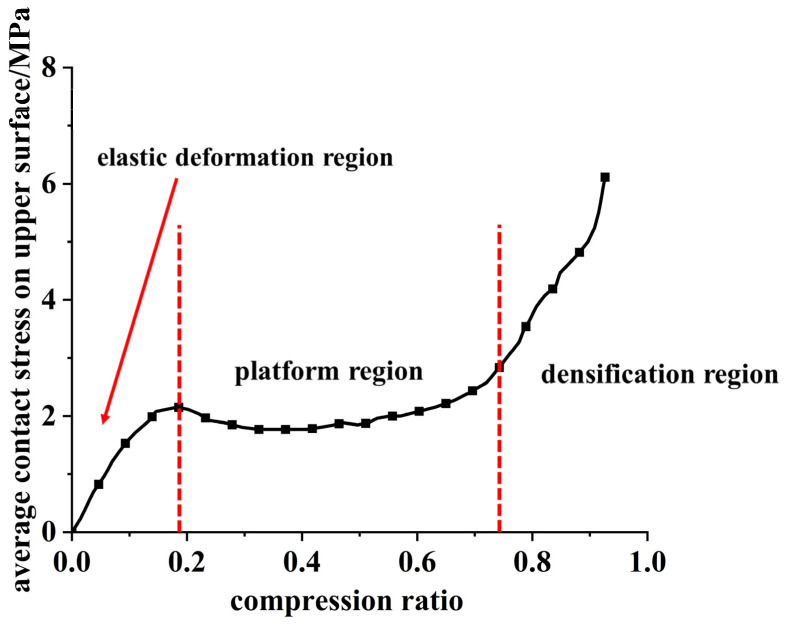
Relationship between average contact stress and compression ratio on upper surface of honeycomb skeleton.

The compressive deformation of a honeycomb skeleton goes through three stages: elastic deformation region, platform region, densification region [33]. Figure 7 shows a function curve of the average contact stress and compression ratio on the upper surface of the honeycomb skeleton under a static compressive load.

It can be seen from the figure that, in the elastic deformation region, the cell wall expanded outward, and the relation between the average contact pressure on the upper surface of the honeycomb skeleton and compression ratio was linear. After entering the platform region, with the increase in the compression ratio, the cell wall folded gradually, and the average contact stress on the upper surface had little variation at this stage. With a further increase in the compression ratio, the whole honeycomb skeleton was crushed, and the hole walls quickly approached and contacted with each other. The average contact stress on the upper surface increased sharply with the further growth in the compression ratio; thus, the process entered the densification region.

**Figure 8:**
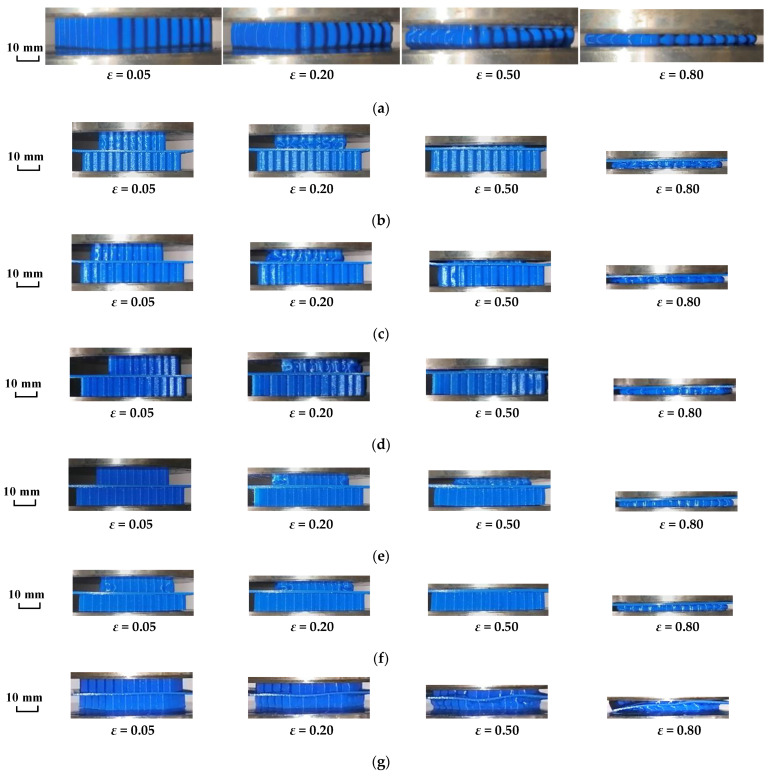

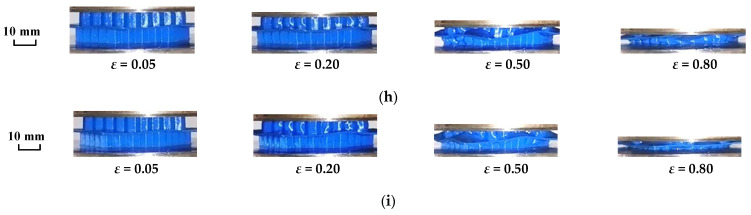
Compression deformation process diagram of each group of honeycomb: (**a**) compression deformation diagram of single-layer honeycomb; (**b**) compression deformation diagram of contraposition tandem honeycomb; (**c**) compression deformation diagram of 3l/4 tandem honeycomb dislocated along *X*-axis; (**d**) compression deformation diagram of 2l/3 tandem honeycomb dislocated along *X*-axis; (**e**) compression deformation diagram of $\sqrt{3}l/4$ tandem honeycomb dislocated along *Y*-axis; (**f**) compression deformation diagram of $\sqrt{3}l/2$ tandem honeycomb dislocated along *Y*-axis; (**g**) compression deformation 10° tandem honeycomb with rotation dislocation; (**h**) compression deformation 20° tandem honeycomb with rotation dislocation; and (**i**) compression deformation 30° tandem honeycomb with rotation dislocation.

Figure 8 is a photograph of the whole process from the compression to compaction of each honeycomb skeleton tandem structure model.

Combined with Figure 6, the whole process of the compressive deformation of the honeycomb skeleton tandem structure was analyzed.

As shown in Figure 8a, the single-layer honeycomb structure underwent buckling from the middle of the cell wall during the compression deformation until it reached the densification stage, while in Figure 6a, it can be seen that the single-layer honeycomb presented a typical compression response curve of the honeycomb structure.

Under the working condition of the translation dislocation of the honeycomb skeleton tandem structure, as shown in Figure 8b–f, the compression deformation first appeared in the upper honeycomb structure, and then the lower honeycomb structure began to show obvious compression deformation after the upper honeycomb structure was compacted. Finally, the whole honeycomb skeleton tandem structure entered the densification stage after the lower honeycomb skeleton was also compacted.

Because there was a certain time difference between the upper and lower honeycomb deformation, two obvious stress platforms can be seen in the response curves in Figure 6a,b. Among them, the 3l/4 tandem honeycomb dislocated along the *X*-axis and $\sqrt{3}l/4$ tandem honeycomb dislocated along the *Y*-axis had obvious compression deformation before the upper honeycomb skeleton was fully compressed, so the second stress platform of the response curve at this dislocation distance was shorter in Figure 6a,b.

When rotational dislocation occurred, as shown in Figure 8g–i, the upper and lower layers of the honeycomb underwent compression deformation at the same time. It can also be seen in Figure 6c that only one obvious stress plateau occurred during the entire response phase. This is due to the fact that, when the yield strength of each layer was different, the layer with the lowest yield strength firstly yielded and destroyed, and then the other layers yielded according to the order of the yield strength from small to large in the compressive process of the honeycomb skeleton tandem structure [34]. It went through three stages: elastic deformation region, platform region, and compaction region. When the honeycomb properties of each layer were identical, each layer yielded at the same time and went through these three stages. In the model of the translational dislocation honeycomb skeleton tandem structure, in order to ensure that the upper honeycomb would not be suspended during the dislocation, the number of cells in the upper honeycomb was less than that in the lower honeycomb, which made the yield strength of the upper honeycomb lower and the yield phenomenon occur first in the upper honeycomb.

## 4. Analysis of Pressure and Energy Absorption Characteristics of Honeycomb Skeleton Tandem Structure

In order to study the mechanical properties and energy absorption characteristics of the honeycomb structures, the average platform stress $\sigma_{p}$ and energy absorption $E_{a}$ of the honeycomb are introduced as evaluation indexes [35]. The average platform stress $\sigma_{p}$ mainly reflects the bearing capacity of the structure, and its calculation formula is:

(4) $$\sigma_{p}=\frac{1}{\varepsilon_{d}-\varepsilon_{c}}\left(\int_{\varepsilon_{c}}^{\varepsilon_{d}}\sigma \left(\varepsilon \right)d\varepsilon \right),$$

where $\sigma \left(\varepsilon \right)$ is the instantaneous stress under strain ε, and $\varepsilon_{c}$ and $\varepsilon_{d}$ represent the starting point and end point of the platform stress stage, respectively.

The energy absorption $E_{a}$ is the absorbed energy during the whole honeycomb deformation process, and its calculation formula is:

(5) $$E_{a}=\int_{0}^{\varepsilon_{d}}\sigma \left(\varepsilon \right)d\varepsilon \times s,$$

where *s* is the equivalent cross-sectional area out of the honeycomb plane.

### 4.1. Analysis of the Influence of Dislocation Mode on the Bearing Performance

**Figure 9:**
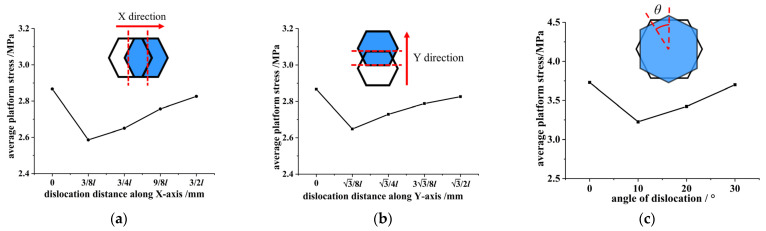
Relationship between average platform stress-dislocation distance/angle of honeycomb skeleton under different dislocation modes: (**a**) dislocation along the *X*-axis; (**b**) dislocation along the *Y*-axis; and (**c**) rotational dislocation.

The average platform stresses of the honeycomb skeleton tandem structures are calculated under different dislocations in Figure 6 according to Formula (2) and plotted in Figure 9.

As can be seen from Figure 9, when the dislocation distance along the *X*-axis increased from 3l/8 to 3l/2, the average platform stress of the honeycomb skeleton tandem structure increased with an enlargement of the dislocation distance. When the dislocation distance was 3l/2, the average platform stress was similar to the ideal contraposition tandem structure. The average platform stress difference between the dislocation distance of 3l/8 and dislocation distance of 3l/2 was 8.8%. When the dislocation distance increased from $\sqrt{3}l/8$ to $\sqrt{3}l/2$, the average platform stress of the honeycomb skeleton tandem structure increased with an enlargement of the dislocation distance. When the dislocation distance was $\sqrt{3}l/2$, the average platform stress was similar to the ideal contraposition tandem structure. The average platform stress difference between the dislocation distance of $\sqrt{3}l/8$ and dislocation distance of $\sqrt{3}l/2$ was 6.4%. When the rotation dislocation angle increased from 10° to 30°, the average platform stress of the honeycomb skeleton tandem structure increased. When the misalignment angle was 30°, the average platform stress was similar to the ideal contraposition tandem structure. The difference in the mean platform stress between the dislocation angle of 10° and dislocation angle of 30° was 13.4%.

In summary, once the tandem structure of the honeycomb skeleton was dislocated, the bearing capacity dropped sharply. However, with an increase in the dislocation distance or dislocation angle, the bearing capacity of the honeycomb skeleton tandem structure was gradually enhanced. The honeycomb skeleton had a periodic topological structure and good symmetry properties. The conditions of dislocation for 3l/2 along the *X*-axis, dislocation for $\sqrt{3}l/2$ along the *Y*-axis, and rotation dislocation for 30° were all half of the period of each dislocation mode of the honeycomb. Therefore, in a dislocation period, when the dislocation reached half of the period, the bearing performance of the honeycomb skeleton tandem structure reached the optimal state, which was similar to that of a contraposition tandem structure.

### 4.2. Analysis of the Influence of Dislocation Mode on Buffer and Energy Absorption Characteristics

**Figure 10:**
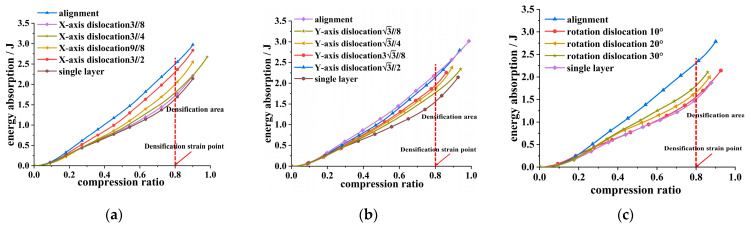
Relationship between energy absorption and compression ratio of honeycomb skeleton under different dislocation modes: (**a**) dislocation along the *X*-axis; (**b**) dislocation along the *Y*-axis; and (**c**) rotation dislocation.

Using Formula (5) to calculate the energy absorption of the honeycomb skeleton tandem structures under various dislocations in Figure 6, we obtained energy absorption curves for the honeycomb skeleton tandem structures under different dislocations, as depicted in Figure 10.

**Figure 11:**
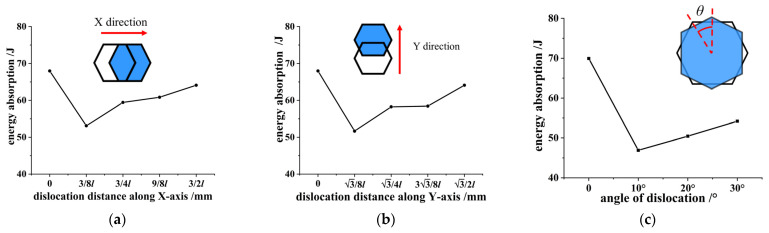
Energy absorption—dislocation distance/angle relationship of honeycomb skeleton under different dislocation modes: (**a**) dislocation along the *X*-axis; (**b**) dislocation along the *Y*-axis; (**c**) and rotation dislocation.

The energy absorption process of honeycomb structures comprises only the elastic deformation stage and platform stress stage, with no consideration given to the densification regions. The platform stress stage is the primary phase for the pressure bearing and energy absorption in honeycomb structures. Figure 11 presents extracted values for the energy absorption in honeycomb structures with different types of misalignments at the end of the platform region shown in Figure 10.

From Figure 11, it is evident that the energy absorption of the honeycomb skeleton tandem structure increased with an increase in the dislocation distance post-dislocation occurrence. In the case of translational dislocations, when the dislocation distance reached half of the dislocation period, the energy absorption capacity of the honeycomb skeleton tandem structure became equivalent to that of a honeycomb structure without any dislocations. On the other hand, in the case of rotational dislocations, the energy absorption of the honeycomb skeleton tandem structure was reduced by 32.97% compared to that of the honeycomb structure without rotational dislocation. The energy absorption of the single-layer honeycomb skeleton was 46.66 J. Compared to the single-layer structure, the tandem honeycomb skeleton exhibited a 31.35% increase in its energy absorption capacity. The optimal performance was achieved when the upper and lower layers were aligned. To ensure the optimal bearing capacity and energy absorption capabilities, the proper rotation displacement must be employed. In product design, it is recommended to align the upper and lower layer arrangement of honeycomb for optimal bearing performance. However, if manufacturing accuracy or product structure limitations arise, utilizing a staggered half of the dislocation period can still yield improved results.

## 5. Influence of Interlayer on Pressure-Bearing Characteristics of Honeycomb Skeleton

The interlayer plays a crucial role in the tandem structure of a honeycomb skeleton, effectively preventing the upper and lower honeycombs from contacting and penetrating each other. This section investigates how the interlayer thickness and material impacted the pressure-bearing characteristics of the honeycomb skeleton tandem structure.

### 5.1. Influence of Interlayer Thickness on Pressure-Bearing Performance

Based on the dislocation model of the honeycomb skeleton tandem structures presented in this paper, we explored the variation law of the pressure-bearing performance by adjusting the thickness of the interlayer. Specifically, we set the thickness to *t*, 1.5 *t*, 2 *t*, 2.5 *t*, and 3 *t*, respectively, where *t* represents the cell wall thickness of the honeycomb skeleton tandem structure.

**Figure 12:**
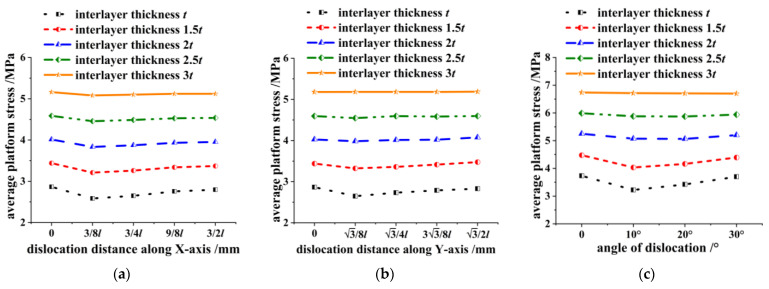
Relationship between average platform stress-dislocation distance/angle of honeycomb skeleton under different interlayer thickness: (**a**) dislocation along the *X*-axis; (**b**) dislocation along the *Y*-axis; and (**c**) rotation dislocation.

It is evident from Figure 12 that the average platform stress of the honeycomb skeleton tandem structure increased as a whole with an increase in the interlayer thickness, while the impact of dislocation on the honeycomb skeleton tandem structure gradually diminished.

This can be attributed to the noticeable trend of the honeycomb walls embedding into the interlayer at the interface of the double-layer honeycomb when compressed. However, the presence of an interlayer resulted in partial deformation resistance and prevented the occurrence of embedding. The thicker the interlayer, the more effective its resistance.

When the interlayer thickness was three times that of the cell wall thickness shown in Figure 12a, both translation and rotation dislocations had no impact on the average platform stress of the honeycomb skeleton tandem structure. Additionally, when the interlayer thickness was twice that of the cell wall thickness shown in Figure 12b, any effect of the dislocation distance on the average platform stress of said structure can be considered negligible. This indicates that the impact of dislocation in the Y direction was inferior to that in the X direction, which aligns with the analysis presented in Figure 9.

### 5.2. Influence of Interlayer Material on Pressure-Bearing Performance of Honeycomb Skeleton Tandem Structure

**Figure 13:**
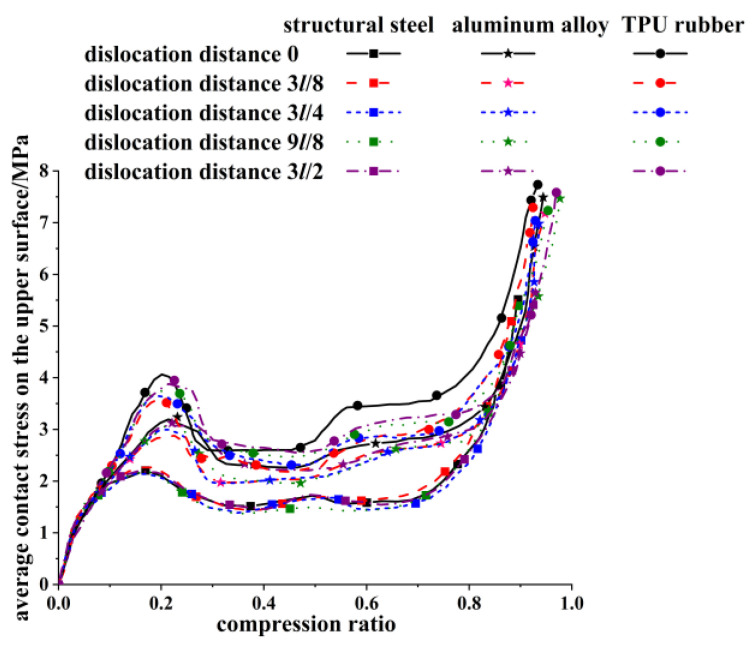
Average contact stress–compression ratio diagram of upper surface of honeycomb skeleton tandem structure with different materials and different dislocation distances along *X*-axis.

The performance of the tandem honeycomb skeleton structure was also influenced by the interlayer material. Different interlayer materials serve different functions for tandem honeycomb skeleton structures [36]. In this section, dislocations along the *X*-axis direction of the honeycomb skeleton tandem structure with interlayers made of structural steel material, aluminum alloy material, and TPU rubber material are simulated, and the influence of the interlayer material on the mechanical properties of honeycomb skeleton tandem structure is analyzed. The average contact stress on the upper surface of various honeycomb skeleton tandem structures was extracted during compression, and is drawn in Figure 13.

**Figure 14:**
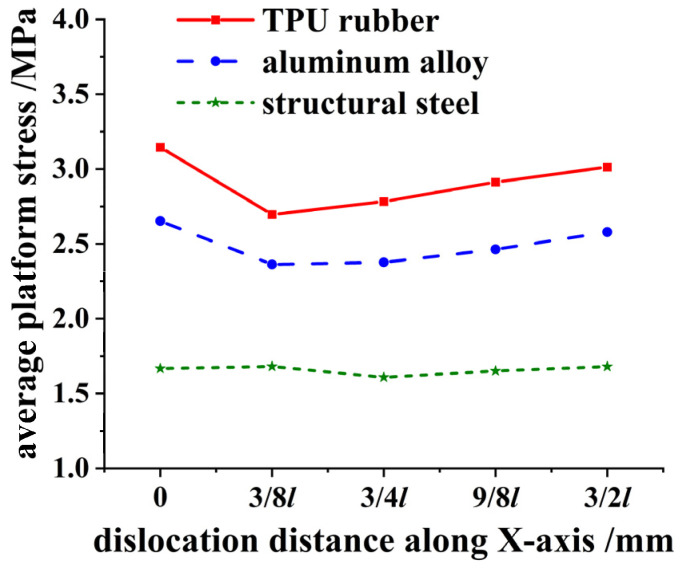
Relationship between average platform stress and dislocation distance of different materials.

The relation curves between the average platform stress values of the honeycomb skeleton tandem structures with different material interlayers along the *X*-axis and dislocation distances are summarized in Figure 13, and the platform stress is extracted in Figure 14.

The bearing capacity of rubber honeycomb skeleton in a tandem structure with a honeycomb arrangement was influenced by the interlayer material. The greater the stiffness of the interlayer, the less sensitive the double-layer rubber honeycomb skeleton was to the dislocation of the upper and lower honeycomb. Meanwhile, by comparing the average contact stresses of the honeycomb skeleton tandem structures with steel and rubber interlayers at different dislocation distances, it can be inferred that a higher stiffness of the interlayer resulted in a lower average contact stress on the upper surface of the platform region in such structures. When utilizing a structural steel interlayer, the average flat stress value of a honeycomb skeleton tandem structure exhibited minimal variation and essentially stabilized at approximately 1.68 MPa. In comparison to a honeycomb skeleton with a rubber interlayer, the average contact stress on the upper surface decreased by 34.2% when employing a structural steel interlayer.

## 6. Conclusions

The different stacking modes between layers affect the mechanical properties and energy absorption properties of tandem honeycomb skeletons. Because rubber materials are prone to deformation, which limits the height of single-layer honeycombs, studying the stacking modes of tandem honeycomb skeletons is crucial for designing rubber honeycomb structures. In the present work, the compressive performance of rubber honeycomb skeleton tandem structures was investigated through quasi-static compression experiments. Compared to that of a single-layer honeycomb skeleton, the energy absorption of a honeycomb skeleton tandem structure could be increased by as much as 1.3 times. The optimal bearing capacity of the honeycomb skeleton was achieved when the upper and lower layers were precisely aligned. Once there existed translation or rotation dislocations between two layers in the honeycomb skeleton tandem structure, both the values of the average platform stress and energy absorption decreased sharply. The bearing capacity of the honeycomb skeleton tandem structures increased with an enlargement of the dislocation (distance or angle), reaching its maximum at the half-dislocation period. Among the translational and rotational dislocations of the honeycomb skeleton tandem structures, the *Y*-axis translational dislocation exerted the least influence. The investigation into the impact of the interlayer partitions suggests that an increase in partition thickness and stiffness led to a reduction in the dislocation-induced effects on the mechanical properties.

Combined with the high viscoelastic properties of rubber materials and the high specific strength advantage of honeycomb skeletons, the rubber honeycomb structure offers more design options for cushioning products. This can be applied to automotive cushions, helmets, knee pads, soles, and other products to ensure energy absorption characteristics while achieving lightweight, soft, and comfortable products.

## Figures and Tables

**Table 1:** Parameters of TPU rubber printing consumables [30].

Diameter of Consumable	Tensile Strength [30]	Bending Strength [30]	Shrinkage Rate STn	Elongation	Melting Point
1.75 mm	450 kg/cm^2^	400 kg/cm^2^	0.8%	170%	190 °C

**Table 2:** Error table of experiment and finite element simulation under different working conditions.

Dislocation Type	Parameter	Error
Dislocation along the	3l/4	5.63%
*X*-axis	3l/2	3.81%
Dislocation along the	$\sqrt{3}l/4$	4.68%
*Y*-axis	$\sqrt{3}l/2$	5.57%
Rotation dislocation	0°	3.34%
	10°	4.61%
	20°	3.7%
	30°	5.21%
Alignment		4%
Single layer		6.21%

## Data Availability

The data presented in this study are available within the article and Supplementary Material.

## Supplementary Materials

The following supporting information can be downloaded at: https://www.mdpi.com/article/10.3390/ma16144933/s1, Data S1: Relationship between average contact stress and compression ratio of upper surface of honeycomb skeleton under different dislocation modes (raw data of Figure 6).
